# Printable nanocomposites of polymers and silver nanoparticles for antibacterial devices produced by DoD technology

**DOI:** 10.1371/journal.pone.0200918

**Published:** 2018-07-19

**Authors:** Nicole Barrera, Lizeth Guerrero, Alexis Debut, Petrus Santa-Cruz

**Affiliations:** 1 Universidad de las Fuerzas Armadas ESPE, Sangolquí, Ecuador; 2 Departamento de Química Fundamental, Universidade Federal de Pernambuco - UFPE, Recife, Pernambuco, Brazil; Institute of Materials Science, GERMANY

## Abstract

Silver nanoparticles (Ag-NPs) are known for their efficient bactericidal activity and are widely used in industry. This study aims to produce printable antibacterial devices by drop-on-demand (DoD) inkjet technology, using Ag-NPs as the active part in complex printable fluids. The synthesis of this active part is described using two methods to obtain monodisperse NPs: chemical and microwave irradiation. The synthesized NPs were characterized by UV-VIS, STEM, TEM, DLS and XRD. Two printable fluids were produced based: one with Ag-NPs and a second one, a polymeric nanocomposite, using silver nanoparticles and polyvinyl butyral (Ag-NPs/PVB). Cellulose acetate was used as a flexible substrate. The ecotoxicity analysis of fluids and substrate was performed with *Artemia franciscana* nauplii. Optimized electric pulse waveforms for drop formation of the functional fluids were obtained for the piezoelectric-based DoD printing. Activity of printed antibacterial devices was evaluated using the Kirby-Bauer method with *Staphylococcus aureus* and *Escherichia coli*. The results show that the printed device with Ag-NP fluids evidenced a bacterial inhibition. An important advantage in using the DoD process is the possibility of printing, layer by layer or side by side, more than one active principle, allowing an interleaved or simultaneous release of silver NP and other molecules of interest as for example with a second functional fluid to ensure effectiveness of Ag activity.

## Introduction

Silver has been used for thousands of years as an antimicrobial and antibacterial agent, up to the antibiotic era which started 90 years ago [1–2]. Nowadays, the dramatic rise of antibiotic-resistant bacteria has led to revisiting the antibacterial activity of silver [3–4]. In the last two decades, nanotechnology has allowed to obtain silver nanoparticles (Ag-NPs) of controlled size and morphology [5]. Silver nanoparticles present a strong broad-spectrum bactericidal activity, and develop little or no bacterial resistance [6,7]. Also, they have the potential to be used alone or in combination with polymers [8]. Other materials are also of interest for such cases. For example, a comparative study of ZnO, CuO and Fe_2_O_3_ nanoparticles activity against Gram-positive and Gram-negative pathogenic bacteria demonstrated that the order of antibacterial activity was ZnO>CuO>Fe_2_O_3_ [9]. A comparison of cytotoxicity of Zn-containing structures showed lower cytotoxic potency of ZnO tetrapods (ZnO-T) than that of ZnO NP, and several parameters were revealed to the assessment of ZnO-T toxicity in cell cultures [10]. The multi-functionality of ZnO is highlighted by Mishra and Adelung (2018) [11], showing both bactericidal activities and biosafe material properties. The low toxicity of ZnO-T, combined with its interconnected porous network present ZnO-T as smart nanocomposites for biomaterials coatings [11]. Regardless nanostructures recently described several pathogenic bacteria have developed resistance against antibiotics and metallic silver, in the form of Ag-NP, remains as an important option as a new generation of antimicrobials. Silver binds to bacterial cell wall and cell membrane and inhibit the respiration process, very likely by the metal interaction with thiol group compounds found in the respiratory enzymes of bacterial cells. In case of *E*. *coli*, silver may act by inhibiting the uptake of phosphate [12]. The large surface/volume ratio of NPs provides better contact, allowing attachment to cell wall, penetration in the bacteria cell, thus, preferably attacking the respiratory chain and leading to cell death. Also, as NP releases silver ions in the bacterial cells, this can enhance their bactericidal activity, by DNA interfering DNA replication after Ag+ contact [13]. The presence of harmful bacteria in water, food and medical equipment has caused the industry to have the need to generate solutions that ensure sterility of products and the prevention of infection [14]. In this field, nanotechnology has been successfully implemented in the industry with the development of processes and products containing nanomaterials, such as the production of Ag-NPs obtained on a large-scale and polymer films, combined or not, to counteract microbial growth [15–16].

There has been an increase in the variety of physical and chemical methods for obtaining silver nanoparticles, leading to new technological applications arising in different areas [17–19], including bioinspired materials as antibacterial devices based on silver nanoparticles [20]. Among the different methods of preparation of Ag-NPs [21–23], we can mention the chemical method that uses the addition of sodium borohydride or sodium citrate and the microwave irradiation method. Silver nitrate is then reduced until obtaining metallic nanoparticles [24–25]. In order to maintain the size and morphology in time of nanoparticles, it is necessary to use stabilizing agents, which immediately wrap them to prevent their continuous growth and agglomeration [26].

To apply these new functional materials by controlled deposition, one of the most innovative techniques to produce template-based devices is the drop-on-demand (DoD), allowing a layer-by-layer production. Inkjet printer system using piezoelectric (PZT) driven printheads may deliver 1 pL drops through an array of nozzles as small as 9 μm. The PZT actuators used in this soft direct-write process do not heat the fluid, avoiding a thermal evolution (thermal reduction, for instance) during the printing process that includes nanomaterials such as Ag-NPs or Ag-NPs polymeric composites. It allows to develop and create new devices that can be applied in different areas such as medicine and food [27–28]. The device properties may also be exploited as a function of the number of printed layers, as for example in “intelligent paper devices” [29], or other nanodevices [30–32].

DoD offers several advantages such as high precision and resolution (ten times higher than the best 3D conventional printer), low sample and reagent consumption, leading to a cost and analysis time reduction [33]. The control of print parameters may be assisted by build-in stroboscopic cameras, and electric pulse waveforms for the drop formation may be obtained for each specific fluid. The final device can be replicated and evaluated. The printing process of nanomaterials encompasses a series of requirements that may be tuned as a function of its composition, allowing a wide range of nanomaterials and even soft materials [34].

As several pathogenic bacteria have developed resistance against antibiotics, bacterial resistance to silver nanoparticles has been discussed in the literature. Panáček et al. reported *Escherichia coli* and *Pseudomonas aeruginosa* resistance to silver nanoparticles after repeated and long exposure, due to the production of an adhesive flagellum protein flagellin, which triggers the NPs aggregation, thus eliminate their antibacterial activity. However, this process can be suppressed in the presence of pomegranate rind extract, which inhibited the production of flagellin protein [35]. One of the advantages of the present proposed system is the novelty of printing more than one active principle, layer by layer or side by side, thanks to the DoD process. In a well-defined architecture template, this would allow an interleaved or simultaneous release of silver and inhibitors of proteins produced by Gram-negative bacteria, that can result in resistance to the antibiotic activity of silver NP.

Each part of the device that will be printed, including functional fluids and substrates, must be evaluated by bioassays to confirm the possible toxicity it may generate on an ecosystem [36]. The effects that are generally evaluated in bioassay are mortality, immobility, behavioral alteration, among others; these allow to observe whether the chemicals or used materials could generate environmental impacts [37]. One of the most common indicator organisms in ecotoxicity tests is *Artemia franciscana* [38–39]. This is a small crustacean that lives in aquatic, high salinity environments. *Artemia franciscana* has been used as a biomodel in preliminary stages of research for new products since 1982 [40–41], and is recommended by FAO [42]. The ability to feed through non-selective filtration makes this micro-crustacean an efficient biosensor for detecting toxicity by filtering large quantities of materials, such as polymeric materials in water [43]. According to the bioassay protocols, to avoid the influence of other factors on their evaluation, *A*. *franciscana* nauplii in their first larval state must remain in optimum conditions of hatching such as temperature, salinity, pH, oxygenation and illumination [44].

Hereafter, the synthesis and characterization of Ag-NPs obtained by chemical method and microwave irradiation, for the creation of two antibacterial devices printed with the DoD technique is described. The first device created uses only Ag-NPs and the second is a polymer nanocomposite made from a mixture of Ag-NPs and polyvinyl butyral. The two devices were printed on acetate cellulose substrate (AC). The ecotoxic evaluation of the two polymers used in the printed device, polyvinyl butyral (in the fluid) and acetate cellulose (substrate) is carried out by studying the mortality of *Artemia franciscana*.

To verify the potential application in a medical environment, antibacterial effects of the devices were evaluated in *Escherichia coli* and *Staphylococcus aureus*, the most prevalent human-associated species of gram-negative and gram-positive bacteria, respectively.

## Materials and methods

### Materials

Silver nitrate (AgNO_3_, ≥99.0%), sodium borohydride (NaBH_4_, 98%) and sodium citrate (C_6_H_5_Na_3_O_7_, ≥99.0%), were purchased from (Sigma-Aldrich, BR). Polyvinyl pyrrolidone (PVP, K90), N,N-dimethylformamide (DMF) and isopropyl alcohol (IA, ≥99.5%) were purchased from (Vetec, BR). Polyvinyl butyral (PVB) was supplied by (Solutia). Acetate cellulose (AC) and Ethanol (Et,≥96%) were supplied by (Dinâmica, BR). Mueller-Hinton (M-H) and agar media were provided by (Difco, BR).

### Synthesis

The active parts of the functionalized fluids to produce the printable antibacterial devices was synthesized using two different process. Chemical synthesis of silver nanoparticles (Ag-NPs (Q)) was performed using the following process: NaBH_4_ (3 mM, 25 mL) was placed in an Erlenmeyer flask under constant stirring, to which AgNO_3_ (1 mM, 10 ml) was added at a rate of one drop per second. C_6_H_5_Na_3_O_7_ (0.05 M, 5 mL) and PVP (3% w/v, 200 μL) were then added at the same speed to stabilize the solution. Microwave radiation synthesis of silver nanoparticles (Ag-NPs (M)) was performed using the following process: DMF (5 mL), AgNO_3_ (4 mM, 1 mL) and PVP (3% w/v, 200 μL) were placed in a test tube, which was placed in the Discover single-mode reactor microwave, CEM brand, at 90°C, 150 W and 250 Psi for 30 seconds with constant magnetic agitation.

### Characterization

The formation of the silver nanoparticles was monitored using UV-Vis spectroscopy. The optical absorption spectra of the Ag-NPs in solution was measured through a spectrophotometer (Analytik Jena SPECORD S 600, DE, using the WinASPECT software). To determine the hydrodynamic diameter distribution of the Ag-NPs, a Dynamic Light Scattering (DLS) analysis was carried out with the filtered samples (0.2 μm filter), using the LB-550 DLS Nanoparticle Size Analyzer (HORIBA, JP). All DLS measurements were performed at a 25 °C fixed temperature. X-ray diffraction (XRD) patterns were collected on an EMPYREAN diffractometer (PANalytical, NL) in Bragg-Brentano configuration at 40 kV and 45 A and monochromatic X-rays of Cu K-α wavelength (λ = 1.541 Å). The analysis was performed from filtered samples, which were previously centrifuged 10 minutes at 10000 rpm, and letting them dry on a microscope slide on a hot plate at 60°C. To analyze the size and morphology of the synthesized Ag-NPs of the filtered samples, images were taken by Scanning Transmission Electron Microscope (STEM), MIRA 3 Field-Emission Gun SEM (Tescan CZ) and Transmission Electron Microscope (TEM), Tecnai G20 Spirit Twin (FEI, NL). The images were analyzed by a software published in [45].

### Ecotoxic analysis of PVB and AC

The ecotoxicity of PVB as a fluid component and AC, as printing support substrate, were analyzed by exposure with *Artemia franciscana*. For the hatching of *A*. *franciscana* cysts, a 35% saline water solution was prepared at pH 8,9. In an Artemio JBL incubator set, 500 mL of the solution was placed with 50 mg of *Artemia franciscana* cysts, maintaining a constant temperature (26°C) and illuminance (1000 lux) for 48 hours, monitored with a luxmeter (WHDZ LX - 1010B). After hatching, the artemias were placed on a 24-well microtitre plate (5 individuals per well) and each well with 1 mL of the previously prepared saline solution.

AC was taken in a layer disc form of 6 mm diameter. PVB was prepared by evaporation, for which 6 mg of PVB was placed in 10 mL of Et and the mixture was done using an ultrasonicator (UNIQUE) for 30 minutes at 50°C. The mixture was then placed on silicone supports in an oven (Technical Novel 512) at 100°C to obtain the polymeric film.

With a metal perforating punch, PVB and AC discs were made. 1, 2 and 3 discs were distributed in different wells, 5 repetitions each. The assay was controlled by two targets in which five individuals were placed. The survival and mortality of *Artemia franciscana* was evaluated at 24 hours, as described in [46].

### Printing procedure for antibacterial printing devices

The antibacterial devices were printed on a Dimatix Materials Printer DMP-2831 (FUJIFILM Dimatix Inc.) using DMC-11610 cartridges (10 picoliters, 16 nozzles) and the jettable fluids were Ag-NPs (Q) and Ag-NPs (M) / PVB (1:1) as a polymer nanocomposite (PNC). In both cases, the printing support was AC.

For each fluid, the drop formation, which is function of the viscosity and surface tension, was optimized varying the electric pulse waveform of the printer. These waveforms, very important mainly for Non-Newtonian fluids, allows the droplets to be ejected properly from the nozzle, carrying the Ag-NPs. The voltage variation (Vxt) to be applied in each PZT actuator was obtained for each fluid with the help of embedded drop watcher and fiducial cameras. DMC-11610 and DMC-11601 are user-fillable 1.5 ml disposable cartridges, that integrates printheads based on PZT silicon single-crystals in micro-electro-mechanical systems (MEMS). They have 16-nozzles of 21 μm or 9 μm diameter respectively, linearly spaced, with drop volume of 10 pL or 1 pL, jetting the fluid in a rate of 5 kHz. Using the Waveform Editor tool embedded in the printer software and the voltage and frequency parameters obtained empirically from a standard fluid, modifications were made to the waveform shape, which influences the formation, shape, and volume of the produced droplets. In order to determine the parameters, the following steps were performed:

1. The DDP applied to the nozzles was scanned between 10–40 V, to determine the minimum DDP for the drop ejection without causing nozzle obstruction and the maximum without generating satellites;
2. The maximum frequency of the jetting cycle (kHz) was then determined avoiding obstruction, generation of satellite drops or jet drift;
3. The Adjustment of the constant DDP plateau was done without fluid flow;
4. The Adjustment of the plateau and filling slopes of the ejection antechamber was done, followed by the same adjustment to increase the DDP applied to the beginning of the ejection of the fluid, and finally for the controlled decrease of the DDP, to guarantee the formation of the drop with the minimum turbulence.

The waveform changes were tracked with the DropWatcher tool camera, while the print parameters were determined from the Drop Spacing tool: to adjust the distance between the center of two adjacent drops, the angle of the print head was adjusted, which influences the resolution of the print.

Although it’s possible to print complex templates, for a Proof of Concept (PoC) purpose device, a basic print pattern (1x1 cm squares) was established to print layer-by-layer assembled devices as a function of the number of layers: 5, 15 or 30, in the present case. The drying time between layer was set to 5 min. Devices were produced with different number of layers for each fluid.

### Analysis of the bactericidal effect with *Escherichia coli* and *Staphylococcus aureus*

Pure strains of *Escherichia coli* (UFPEDA 224) and *Staphylococcus aureus* (UFPEDA 02) were used for bacteriological analysis. The inocula were adjusted to 0.5 on the McFarland scale. A 6.5% w/v solution of the M-H medium with constant agitation at 60°C was prepared for the culture medium and 20 g of agar was then added. The mixture was autoclaved and placed in Petri boxes. The bacteria were sown in the Petri dish boxes with a swab over the entire surface, on which the discs of printed devices (5,15 and 30 layers) were placed. The discs were prepared for this assay using a standard metal perforating punch. As target control, sterile AC discs were used. The boxes were incubated at 37°C for 24 hours and the inhibition zone was then measured with a digital caliper (Mitutoyo). To insert the Ag-NPs, a well has been made in the center of the Petri dish, drilling the middle and removing it with the help of a cork drill. The diffusion coefficient of Ag-NPs in the M-H-agar medium was calculated by measuring the apparent dynamic viscosity of the culture medium in the following concentrations: M-H (0.50% w/v) and agar (0.15% w/v), M-H (1.00% w/v) and agar (0.31% w/v), M-H (1.50% w/v) and agar (0.46% w/v). The data were then extrapolated to determine the apparent viscosity at the concentration M-H (6.50% w/v) and agar (2.00% w/v) used in the bactericidal effect analysis. The diffusion coefficients (D) were determined with the Stokes-Einstein equation: $D=\frac{k.T}{3.\pi .\eta .d(H)}$ (herein nm^2^/s), where *k* is the Boltzmann constant, *T* the absolute temperature, *η* the dynamic viscosity and d(H) the hydrodynamic diameter. Finally, the measured inhibition zone was related to the minimum inhibition zone *A*_*min*_ expected in relation to the diffusion coefficient calculated in 24 h of bacterial incubation, as indicated in the following relation: $A_{min}=\sqrt{D\times 24h}$ (herein mm^2^).

### Statistical analysis

Survival of *Artemia franciscana*: statistical analysis was performed using an ANOVA variance analysis of a 2x3 factorial design (6 treatments) to evaluate the combination of two types of levels: material type (PVB and AC) and concentrations of each (0.4,0.8,1.2 mg/mL for PVB and 1,2.3 mg/mL for AC) using the IBM SPSS statistical program. A *p* value ≤ 0.05 was considered.

Bacterial inhibition: Two analyses of variance (ANOVA) were performed for a factorial 2x3 (6 treatments) design. One for the Ag-NPs (Q) printed device and one for the Ag-NPs (M) / PVB printed device. In both analyses the combination of bacterial type (*E*. *coli* and *S*. *aureus*) and number of printing layers (5,15 and 30) was evaluated by the IBM SPSS statistical program. A *p* value ≤ 0.05 was considered. For each experimental measurement, average value and standard deviation were calculated for the inhibition zone using a millimeter ruler.

## Results and discussion

### Ultraviolet–visible spectroscopy

**Fig 1:**
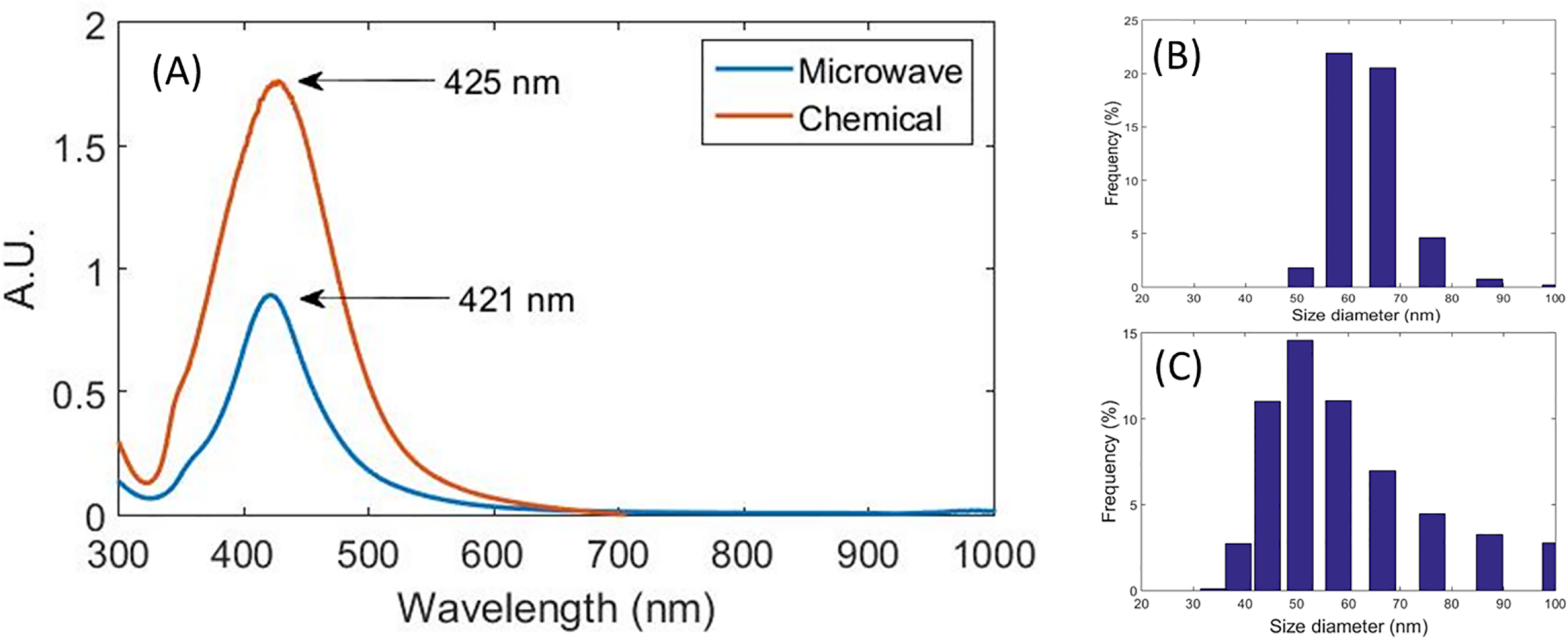
UV-VIS spectrum. (A) and DLS measurements for silver nanoparticles produced by microwave (B) and chemical (C) synthesis.

The presence of silver nanoparticles was confirmed both in the chemical synthesis method with a maximum absorption peak at 425 nm and in the microwave irradiation method with a peak at 421 nm (see Fig 1A). This indicates an approximate size for spherical silver nanoparticles between 35 to 50 nm [47]. This conclusion is based on the size dependence of the optical property of the plasmon resonance of silver nanoparticles [48]. The frequency of the surface plasmon resonance over the silver nanoparticles depends on the nanoparticle shape and size, as well as the dielectric functions of the host matrix and nanoparticle. Also, the formation of nanoparticles aggregates or clusters may displace the absorption band up to the infrared, not observed in the present work [49].

### Dynamic Light Scattering

The hydrodynamic diameters of the nanoparticles were determined using DLS. In cases of polydisperse size distribution of nanoparticles, the greater the dispersity of the sample, the greater the probability of unsuccessful data from DLS technique. The average diameter was found to be 59.59 ± 15.8 nm for Ag-NPs (Q), and 64.29 ± 8.62 nm for Ag-NPs (M), as shown in Fig 1B and 1C.

### X-ray diffraction

**Fig 2:**
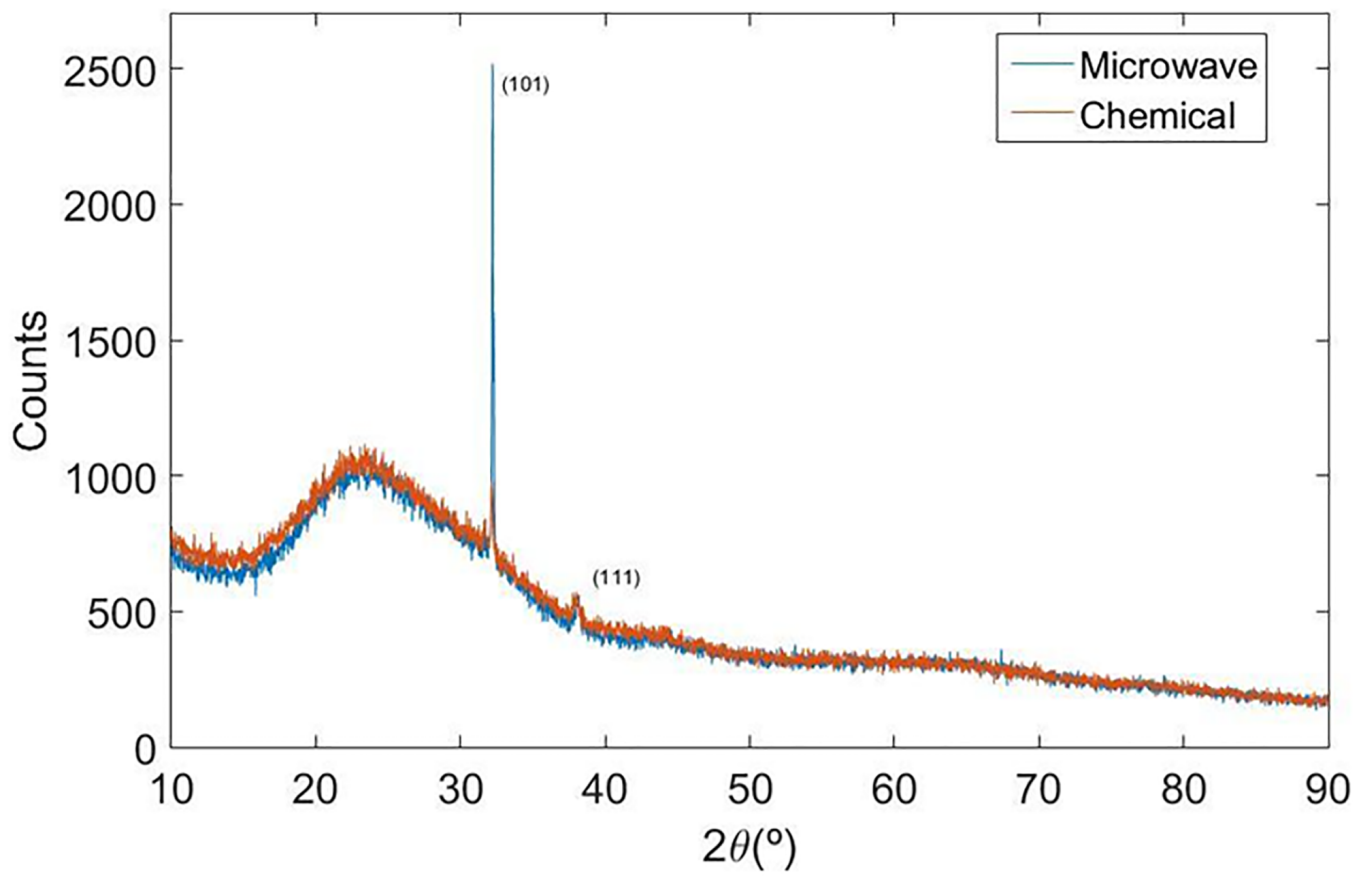
XRD patterns from Ag-NPs produced by microwave and chemical preparation.

From Fig 2, diffracted peaks were observed at 32.12° and 38.07° for Ag-NPs (Q). They correspond to the set of planes (101) and (111) of the crystalline structure of silver (FCC). For Ag-NPs (M), the diffraction angles 32.12° and 38.06° correspond to the (101) and (111) planes, respectively [50–52]. As shown in the diffractogram of Fig 2, one may notice that, regardless of the nanoparticle preparation technique (microwave or chemical), the ratio between the Bragg peaks intensities from the metallic silver and the scattering band associated to the amorphous part of the material, do not changes, although the intensity ratio between the peaks related to (101) and (111) planes have its ratio intensities changed, probably due to a possible preferential orientation of the powders during the preparation of the samples.

### Scanning Transmission Electron Microscope (STEM) and Transmission Electron Microscope (TEM)

**Fig 3:**
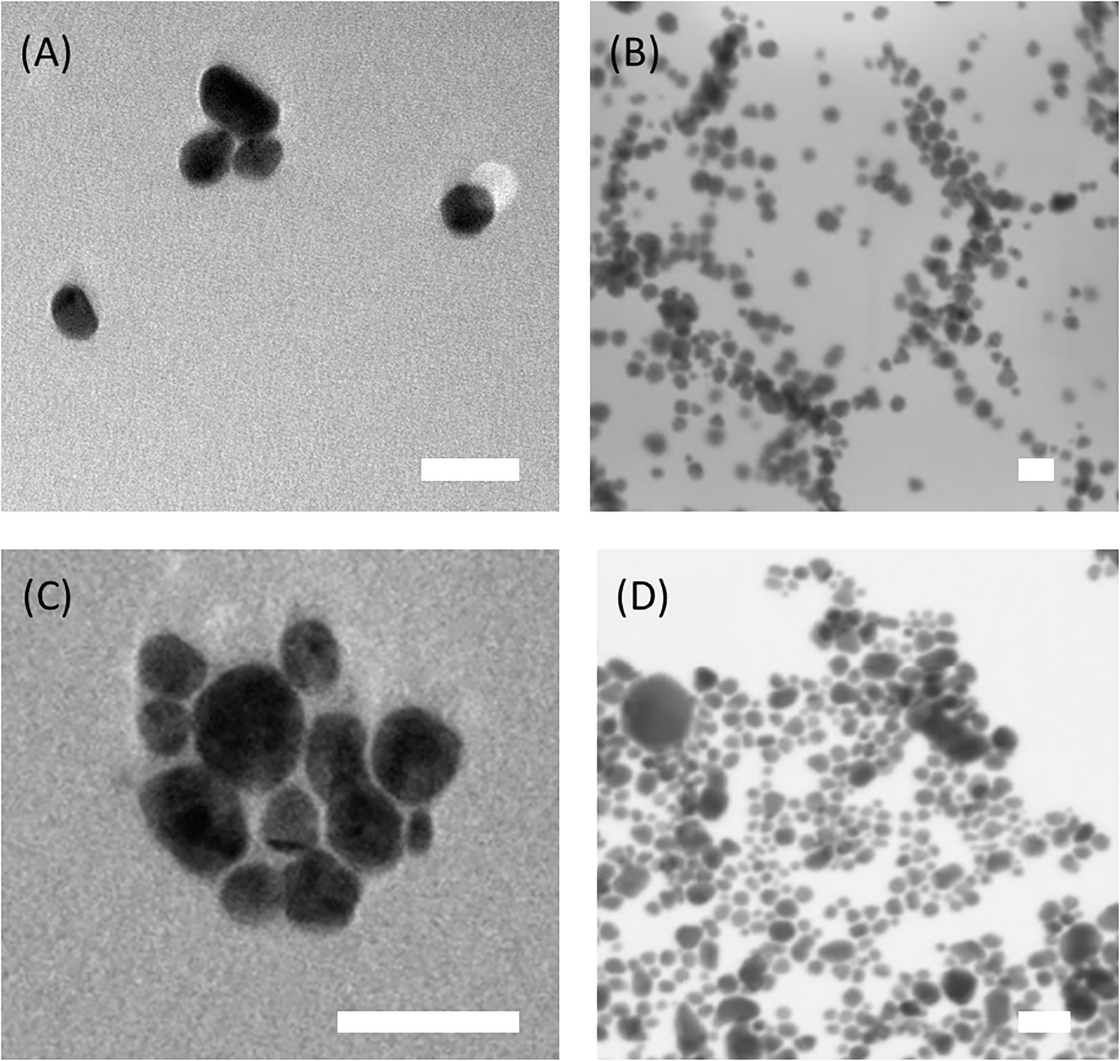
TEM and STEM micrographs of silver nanoparticles. Prepared from microwave synthesis (A) and (B), and from chemical synthesis (C) and (D). The white bar scale is 50 nm.

From Fig 3, one can observe that Ag-NPs (Q) and Ag-NPs (M) are spherical. Using an analysis software developed in our group [45], an average diameter of 18.9 ± 12.3 nm was found for Ag-NPs (Q), taking 132 nanoparticles into consideration. For Ag-NPs (M) an average diameter of 17.83 ± 7.3 nm was found measuring an amount of 113 nanoparticles. This result is different from the DLS measurements, as the refraction index of the formed complex is unknown and due to the fact that the sample distribution is not perfectly monodisperse [3].

### Ecotoxic analysis of PVB and AC

In this research a range of 0 to 10% of *A*. *franciscana* mortality was used to determine the non-toxicity of the polymer, based on the results obtained by Chinnasamy et al. [53]. In addition, an experimental error rate of 5% was established. To verify this experimental error rate, *A*. *franciscana* nauplii were exposed to PVB. A mortality of 4% against exposure of 0.4 mg/ml and 8% against exposure of 0.8 and 1.2 mg/ml was obtained, lower than the established experimental error. For individuals exposed to AC, an 8% mortality was observed at exposure of 1 mg/ml AC and a 12% mortality at exposure of 2 and 3 mg/ml AC, lower than the considered experimental error. Therefore, comparing the survival rate of control individuals with respect to individuals exposed to PVB and AC, we conclude that there is no influence of these polymers on the survival of *A*. *franciscana*.

### Printed devices and analysis of the bactericidal effect with Escherichia coli and Staphylococcus aureus

**Fig 4:**
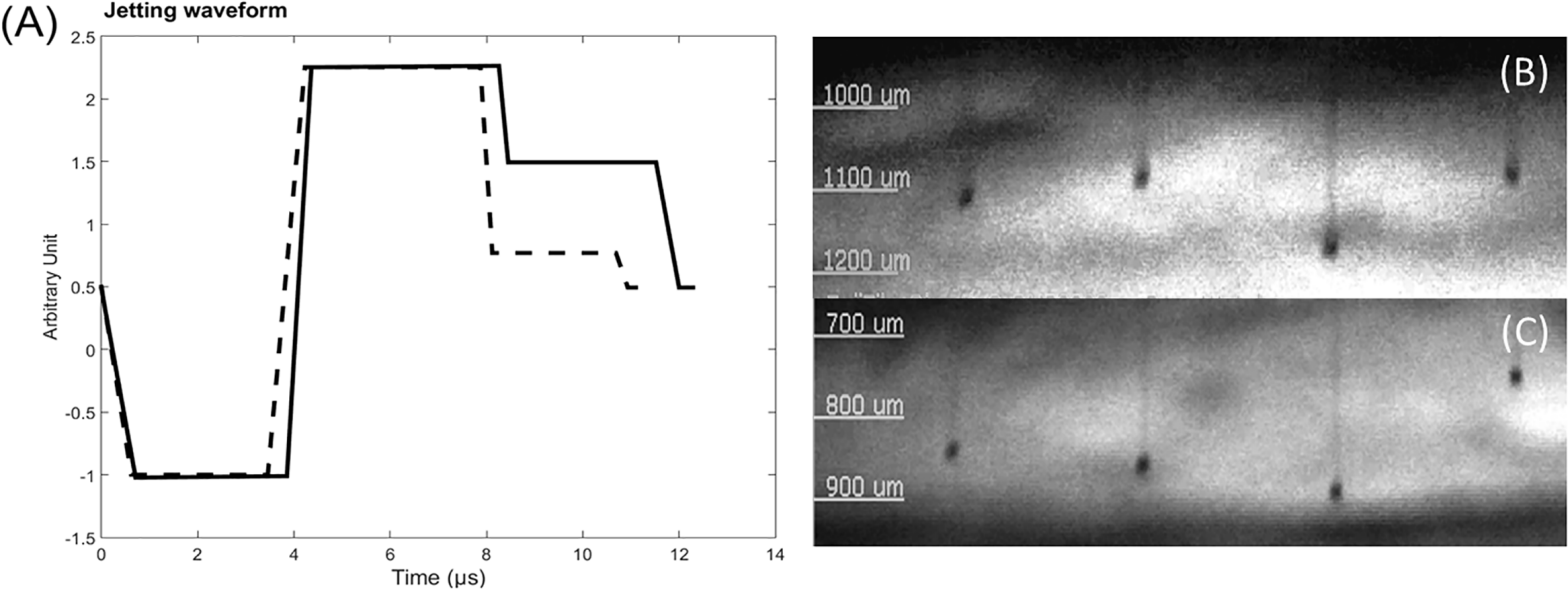
Waveform curves (Vxt). Vxt optimized for Ag-NPs (M) / PVB (A) and Ag-NPs (Q) (B) -based fluids (left) and the corresponding quick shot images of the jetted droplets showing six drops (of 16) from the DoD printer at final stages of drop formation (right).

To produce the two printed devices, the fluids droplets ejected through the nozzles were optimized applying a specific electric pulse waveform to the PZT actuators. Table 1 and Fig 4 (left) show the four-segment control settings and the corresponding waveform curves (Vxt) for printing Ag-NPs (M) / PVB and Ag-NPs (Q), and the corresponding quick shot images of the jetted droplets is shown beside (Fig 4, right). A detailed protocol can be found in [54].

**Table 1:** Four-segment control settings for the processing of calibration curves for printing Ag-NPs (M) / PVB and Ag-NPs (Q).

	Segment Control	Level %	Decay	Span Time (μs)
**Ag-NPs (M) / PVB**	A	0	0.40	3.586
	B	93	1.05	4.864
	C	67	0.59	3.392
	D	40	0.73	0,768
**Ag-NPs (Q)**	A	0	0.55	3.456
	B	87	0.90	4.416
	C	67	0.60	2.816
	D	40	0.73	0.768

**Fig 5:**
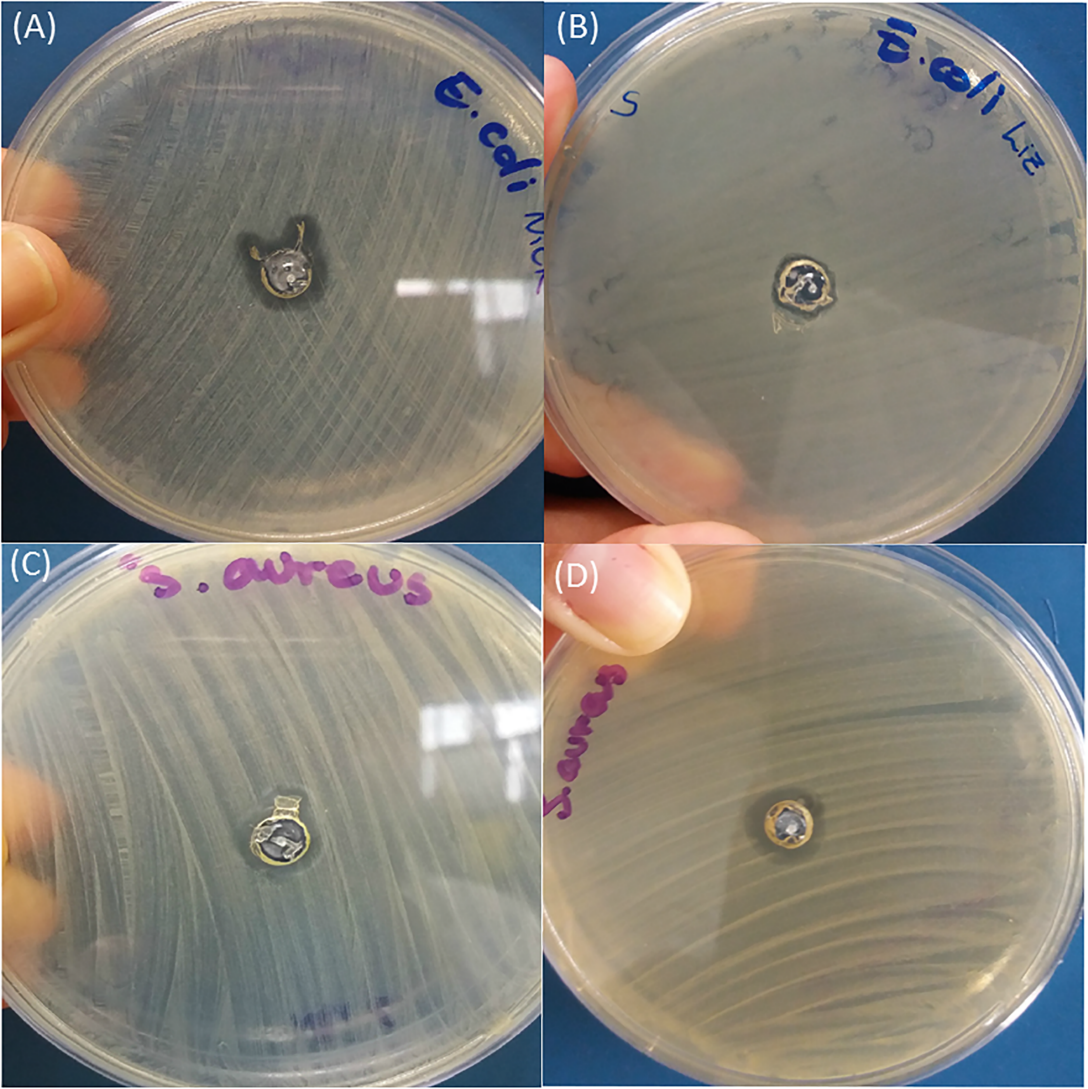
Bactericidal effect on *E*. *coli*. (A) Ag-NPs (Q), (B) Ag-NPs (M) and on *S*. *Aureus* (C) Ag-NPs (Q), (D) Ag-NPs (M).

In order to evaluate the efficacy of the printed antibacterial devices, initially the bactericidal effect of silver nanoparticles without PVB and before printing was observed for Ag-NPs (M). The effect was compared between Ag-NPs (Q) and Ag-NPs (M) after incubation of *E*. *coli*, shown in Fig 5A(a) and 5B, respectively, and with *S*. *aureus*, shown in Fig 5C and 5D, respectively.

**Fig 6:**
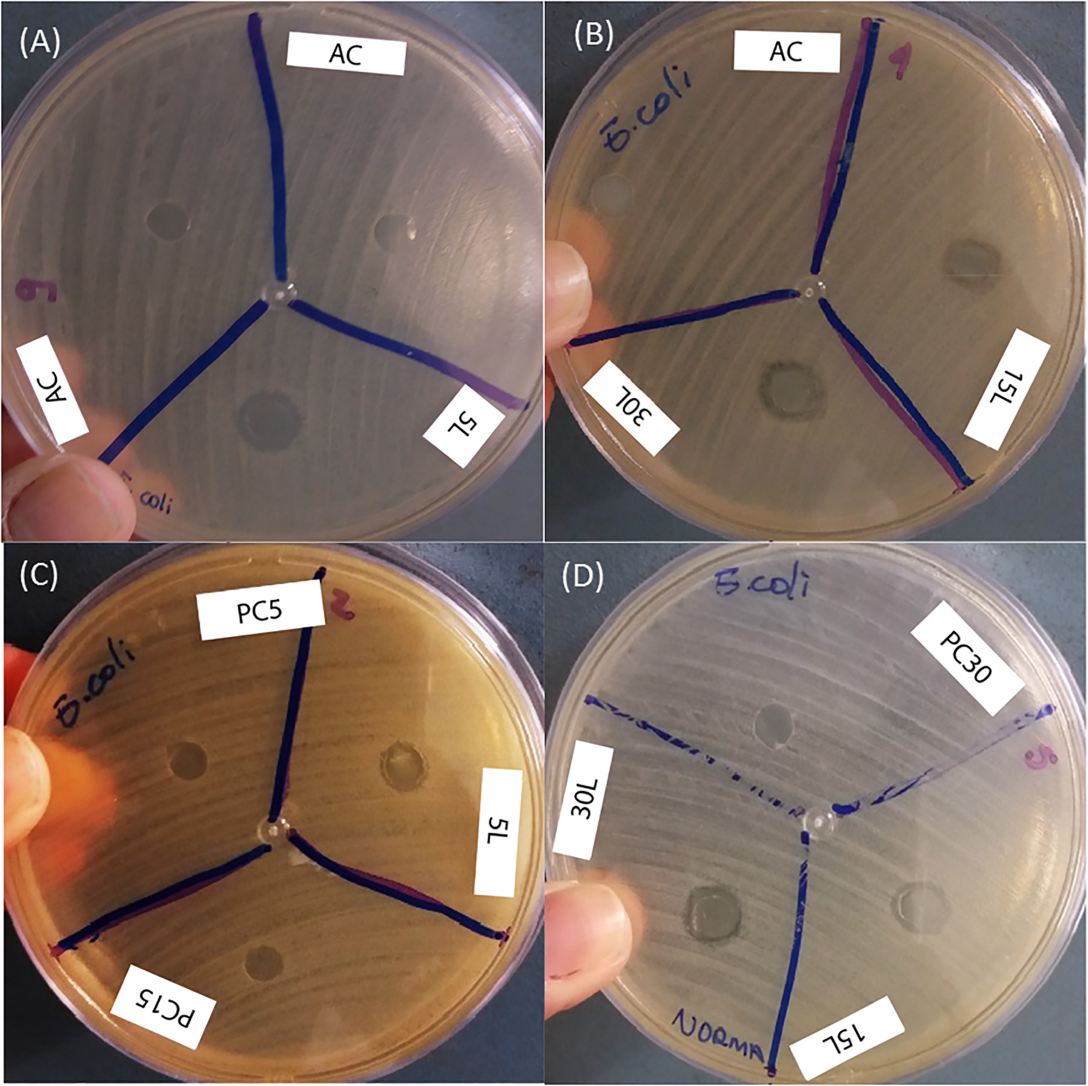
Bactericidal effect on *E*. *coli*. (A) Ag-NPs (Q) on AC and 5 layers (5L), (B) Ag-NPs (Q) on AC and 15 and 30 layers (15L and 30L), (C) Ag-NPs (M)/PVB on 5 layers (5L), (D) Ag-NPs (M)/PVB on 15 and 30 layers (15L and 30L).

**Fig 7:**
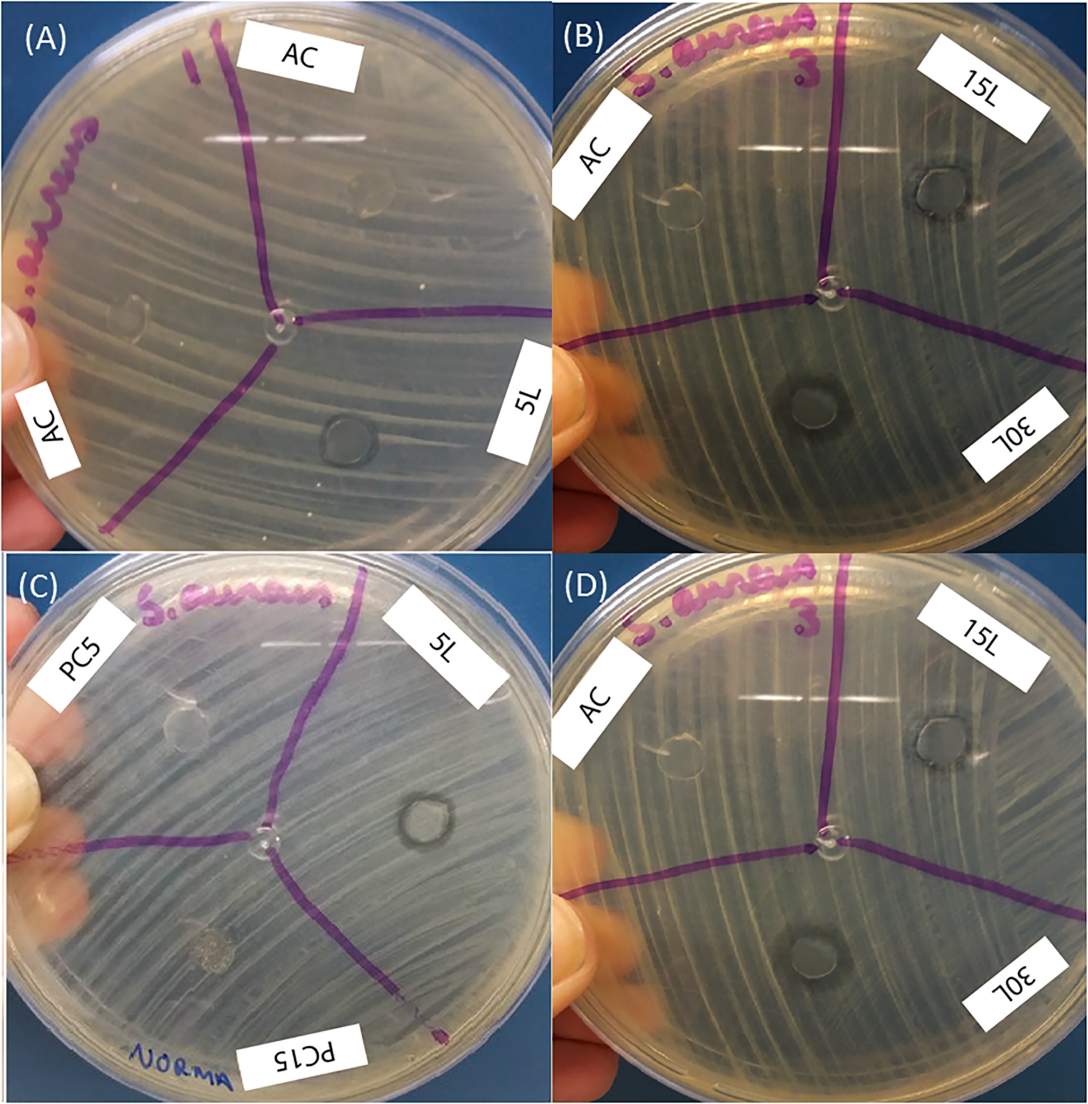
Bactericidal effect on *S*. *aureus*. (A) Ag-NPs (Q) on AC and 5 layers (5L), (B) Ag-NPs (Q) on 15 and 30 layers (15L and 30L), (C) Ag-NPs (M)/PVB on AC and 5 layers (5L), (D) Ag-NPs (M)/PVB on 15 and 30 layers (15L and 30L).

After the production of the printed devices, the bactericidal effect was analyzed using the method of diffusion in agar with Kirby—Bauer discs, where the inhibition zone generated by the devices of Ag-NPs (Q) and Ag-NPs (M) in both bacteria was evidenced. The results for the AC (substrate) and the PVB polymer printed without Ag-NPs (called PC5, PC15 and PC30 in the figures) were used as controls. There were compared with the nanoparticle-printed devices, where a higher inhibition zone was observed in the Ag-NPs (M) / PVB devices, as shown in Fig 6. Measurements were registered (Table 2) for the inhibition zones produced by devices printed with Ag-NPs (Q) with 5 (Fig 6A), 15 and 30 (Fig 6B) printed layers, and devices with Ag-NPs (M) with 5 (Fig 6C), 15 and 30 (Fig 6D) printed layers, on *E*. *coli*. The same process of measurements was performed for *S*. *aureus* (Fig 7). All the inhibition zones measurements are summarized in Table 2.

**Table 2:** Inhibition zone size in mm for Ag-NP (Q) and Ag-NP (M) / PVB devices.

**Ag-NPs (Q)**			
**Numbers of layers**	**Average value of the inhibition zone (mm)**		**Diffusion coefficient (nm**^**2**^**/s)**
	***E*. *coli***	***S*. *aureus***	
A5	0.99 ± 0.62	0.25 ± 0.14	1.91x10^4^
A15	1.65 ± 0.97	0.55 ± 0.25	
A30	1.68 ± 0.96	1.24 ± 0.70	
**Ag-NPs (M) / PVB**			
**Numbers of layers**	**Average value of the inhibition zone (mm)**		**Diffusion Coefficient (nm**^**2**^**/s)**
	***E*. *coli***	***S*. *aureus***	
A5	1.07 ± 0.49	0.33 ± 0.17	1.76x10^4^
A15	1.79 ± 1.02	0.62 ± 0.28	
A30	1.93 ± 0.97	1.59 ± 0.78	

By the previous results, one can conclude that the cell wall thickness of the Gram-positive bacteria affects the action of the devices, showing less effect in *S*. *aureus* with respect to *E*. *coli*. This is also confirmed by other research groups, as published by Malegowd et al. [55]. Diffusion coefficients were determined for Ag-NPs (Q) and Ag-NPs (M), see Table 3.

**Table 3:** Diffusion coefficients of synthesized Ag-NPs in Mueller-Hinton culture medium.

Diffusion coefficients		
DLS average diameter of Ag-NPs (nm)	Equation	Diffusion coefficients (nm^2^/s)
**Ag-NPs (Q)** d(H) = 80.97	$D=\frac{k.T}{3.\pi .\eta .d(H)}$ k = 1.38064852 x 10^−23^ J.K^-1^ T = 310.15 K *η* = 2.926x10^-28^ J.s/nm^3^	D = 19177
**Ag-NPs (M)** d(H) = 87.84		D = 17677

**Fig 8:**
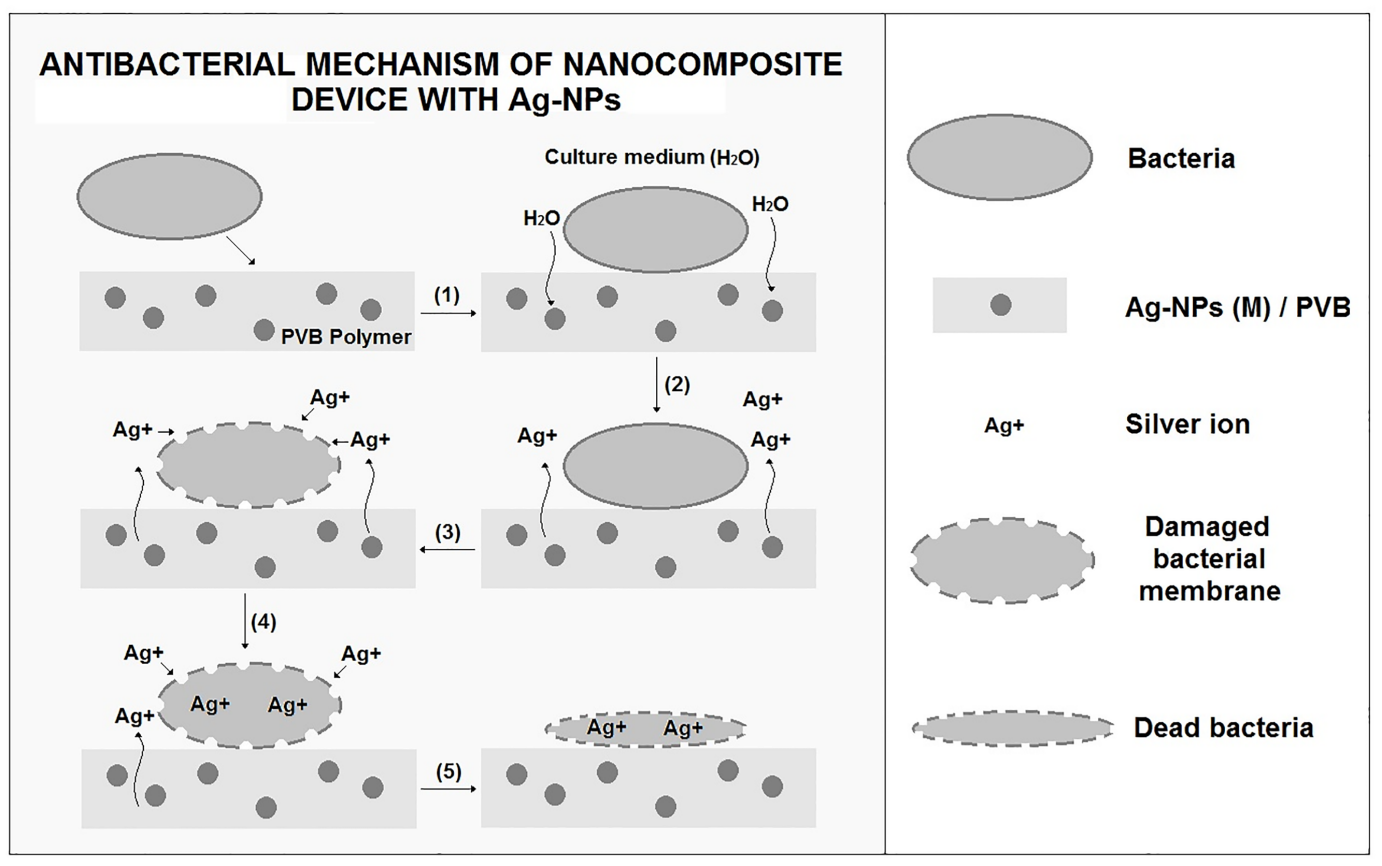
Schematic diagram of antibacterial mechanism of the device impregnated of AgNPs.

Furthermore, it was measured an apparent minimum inhibition zone for Ag-NPs (Q) of 0.041 mm and 0.039 mm for Ag-NPs (M) / PVB. These experiments show clearly the antibacterial effect of the obtained devices. However, the antibacterial mechanism of Ag-NPs is not clearly defined. There are several theories in the current literature. Ag-NPs can anchor to the cell membrane, causing damage and leakage of intracellular material. The theory mentions that the formation of free radicals by Ag-NPs is one of the causes of cell death since they have the ability to generate pores in the membrane [56]. Another theory considers that the release of silver ions from nanoparticles damages the integrity and permeability of the membrane, and that these ions can react with functional protein molecules and DNA, interfering with DNA metabolism and replication, originating the cell death [25]. Considering these various theories, we consider the schematic diagram for explaining the antibacterial mechanism presented here in the nanocomposite system, see Fig 8. The bacteria interact with the composite and in presence of water a process of creating silver ion is generated, thus leading to the antibacterial effect. The mechanism presented in Fig 8 is related to metallic pure silver nanoparticles, that releases ionic silver in aqueous solution, as proposed here, but, using the same procedure proposed in the present paper, it is also possible to print new hybrid structures, as those recently proposed by Assis et Al. [57]. These hybrid nanoparticles act by a different mechanism, in which a semiconductor attracts bacterial agents and Ag nanoparticles neutralize them, in α-Ag2WO4 nanoparticles, produced by femtosecond laser irradiation. The new class of spherical hybrid nanoparticle presents a 32-fold improvement of antibacterial performance and may be fully compatible with the presented DoD printing process.

### Statistical analysis

The result of the ANOVA, carried out in the survival of *Artemia franciscana*, showed that there is no influence of the polymer type (user in fluid and substrate), since the *p* value obtained for the type and the concentration of each polymer is 0.865 and 0.523, respectively. Moreover, these values are greater than 0.05. So, with a 95% confidence interval, we determined that there is no statistically significant influence of this parameter against the microcrustaceans taken as a biomodel. Regarding the ANOVA analysis on bacterial inhibition, maintaining a 95% confidence interval (*p*_value 0.05), we determined that the bacterial type has a statistically significant influence on this parameter, since the *p*_value resulting from the analysis was 0.028. However, no significant influence related to the number of printing layers on bacterial inhibition was found, since the calculated *p*_value was 0.15.

## Conclusions

Two antibacterial devices printed using DoD technology were obtained. The first, with silver nanoparticles obtained by chemical process and the second one, with silver nanoparticles produced under microwave irradiation, in a PVB composite. We show that both devices have a bactericidal effect against *S*. *aureus* and *E*. *coli*. The substrate (acetate cellulose) and the polymer used in the fluid (PVB) were subjected to an ecotoxic test, in which it was determined that both polymers do not generate any type of toxicity, according to the biomodel used. The results indicate that the best antibacterial characteristics of the printed device is obtained for the polymer compound with silver nanoparticles synthesized by microwave irradiation and considering the highest number of printing layers. In addition, the inclusion of silver nanoparticles into the polymer matrix avoid any aggregation and so the possibility of a reduction of its antibacterial activity. To ensure the effect on a long time, an interleaved or simultaneous release of silver and inhibitors of proteins produced by Gram-negative bacteria could be produced in the next future. Therefore, this kind of device could be used for industrial applications as a printable antibacterial device produced by drop-on-demand inkjet technology.

## Supporting information

S1 Fig Original print screen of waveform curves (Vxt). Vxt optimized for Ag-NPs (M) / PVB.
(JPG)

S2 Fig Original print screen of waveform curves (Vxt). Vxt optimized for Ag-NPs (Q).
(JPG)

S1 Video Droplet video obtained during the printing process using parameters defined in S1 Fig. (MP4)

S2 Video Droplet video obtained during the printing process using parameters defined in S2 Fig. (MP4)
