# Supplementary figures and images for: Printable nanocomposites of polymers and silver nanoparticles for antibacterial devices produced by DoD technology
Source: PLoS One. 2018 Jul 19;13(7):e0200918. doi: 10.1371/journal.pone.0200918 (PMC6053237; doi:10.1371/journal.pone.0200918)

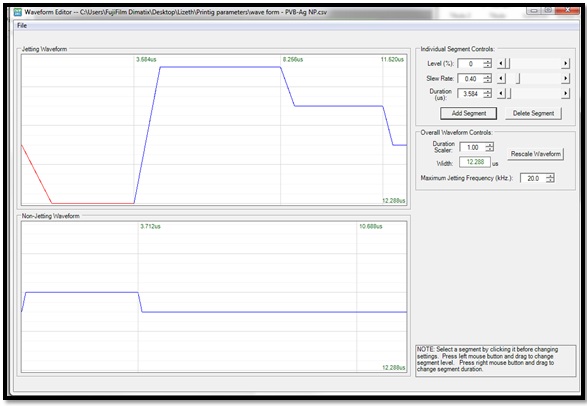

Supplement: S1 Fig — Vxt optimized for Ag-NPs (M) / PVB. (JPG) [file pone.0200918.s001.jpg]

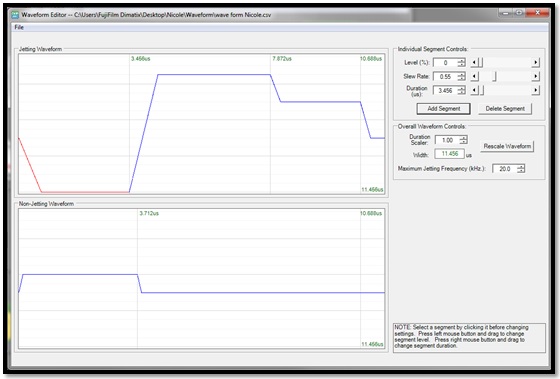

Supplement: S2 Fig — Vxt optimized for Ag-NPs (Q). (JPG) [file pone.0200918.s002.jpg]
